# The preclinical analysis of TW-37 as a potential anti-colorectal cancer cell agent

**DOI:** 10.1371/journal.pone.0184501

**Published:** 2017-10-24

**Authors:** Shun Lei, Yao Ding, Yun Fu, Shuang Wu, Xiong Xie, Cancan Wang, Houjie Liang

**Affiliations:** 1 Department of Oncology and Southwest Cancer Center, Southwest Hospital, the Third Military Medical University, Chong Qing, China; 2 Department of General surgery, Gao Xin District People’s Hospital of Chong Qing, Chong Qing, China; Suzhou University, CHINA

## Abstract

TW-37 is a novel, potent and non-peptide Bcl-2 small-molecule inhibitor. Its activity in colorectal cancer (CRC) cells is studied. In both HCT-116 cells and primary human colon cancer cells, treatment with TW-37 at only nM concentration efficiently inhibited cell survival and proliferation. TW-37 also induced caspase-3/9 and apoptosis activation in CRC cells. Feedback autophagy activation was observed in TW-37-treated CRC cells. Reversely pharmacological autophagy inhibition or Beclin-1 knockdown by targeted-shRNA potentiated TW-37-induced apoptosis and killing of CRC cells. *In vivo*, intravenous injection of TW-37 inhibited HCT-116 tumor growth in mice. TW-37’s anti-tumor activity was further potentiated against Beclin-1-silenced HCT-116 tumors. Together, targeting Bcl-2 family protein by TW-37 efficiently inhibits CRC cell growth *in vitro* and *in vivo*. Inhibition of feedback autophagy activation could further sensitize TW-37.

## 1. Introduction

Colorectal cancer (CRC) is the third most-common human malignancy[1,2,3,4]. Each year, it is estimated that over one and half million new cases of CRC will be diagnosed [1,2,3,4]. The current CRC treatment options include the combinations of surgery resection, chemo-therapy and/or radio-therapy[5,6]. However, for the patients with advanced, recurrent or metastatic CRC, the prognosis is often poor [5,6].

The Bcl-2 family proteins play a pivotal role in the regulation of cell apoptosis and proliferation[7]. There are two major categories of Bcl-2 family proteins, including the anti-apoptosis proteins (Bcl-2, Bcl-xL, Bcl-w, and Mcl-1), and the pro-apoptotic proteins (Bim, Puma, Bid, Bad, Bax, Bak and others)[7]. The anti-apoptosis Bcl-2 proteins contain four BH regions (BH1–BH4), and the pro-apoptotic Bcl-2 proteins are BH3-only proteins [7]. Tumors (*i*.*e*. CRC) expressing high levels of Bcl-2, Mcl-1, or Bcl-xL are often resistant to chemotherapy and radiotherapy [8,9].

Recent studies have developed TW-37 as a novel, potent Bcl-2 small-molecule inhibitor [10,11,12,13]. It inactivates multiple anti-apoptotic Bcl-2 family proteins[10,11,12,13]. TW-37 binds directly to BH3 domain-Bcl-2 family proteins to shut down the hetero-dimerization of pro-apoptotic proteins (Bid, Bim, and Bad) with Bcl-2. It therefore prevents cell apoptosis [10,11,12,13]. The pre-clinical cancer studies have suggested that TW-37 could inhibit cancer cells by inducing cancer cell apoptosis[10,11,12,13]. The current study examined its activity against human CRC cells.

## 2. Materials and methods

### 2.1. Chemicals and reagents

TW-37 was provided by Selleck LLC (Nanjing, China). Three-methyladenine (3-MA) and chloroquine (Cq) were obtained from Sigma (Shanghai, China). All the antibodies in this study were purchased from Cell Signaling Tech (Shanghai, China). Puromycin was also purchased from Sigma (Shanghai, China). The medium an reagents for cell culture were provided by Gibco (Shanghai, China).

### 2.2. Primary culture of human cells

A total of two colon cancer patients (Both female, 56/61 years old, undergoing whole colon cancer resection), administrated at Southwest Hospital, the Third Military Medical University, were enrolled in this study. The fresh colon cancer tissues and surrounding normal epithelial tissues were separated very carefully under the microscopy. Tissues were then minced, washed, and digested by collagenase I (Sigma). Digestions 2–5 were neutralized, pooled, and filtered. Single cell suspensions of primary human colon cancer cells and colon epithelial cells were cultured in the described medium for primary human cells [14,15]. Two primary human colon cancer cell lines and one primary colon epithelial cell line were established in this study. The protocols using human tissues were conducted according to the principles of Declaration of Helsinki, and were approved by the Ethics Review Board (ERB) of The Third Military Medical University. Written-informed consent was obtained each participant.

### 2.3. Culture of HCT-116 cells

HCT-116 cells were provided by Dr. Lu[16]. Cells were cultured in the RPMI 1640 medium plus 10% fetal bovine serum (FBS), in regular CO_2_ incubator.

### 2.4. MTT assay

The routine MTT (Sigma) dye assay was performed to test cell viability. MTT optical density (OD) value at 590 nm was recorded.

### 2.5. Colony formation assay

Following the TW-37 treatment, HCT-116 cells were re-suspended in complete medium with 0.5% of agar (Sigma), which were then added on the top of a pre-solidified six-well plate. TW-37-containing medium was renewed every two days. After 8 days incubation, the number of surviving colonies was counted.

### 2.6. BrdU incorporation assay of cell proliferation

HCT-116 cells were initially plated into 96-well plate (1 ×10^4^ cells/well). After the indicated TW-37 treatment, HCT-116 cells were further incubated with BrdU (10 μM, Cell Signaling Tech, Shanghai, China) for additional 12 hours. Cells were then fixed, and BrdU incorporation was determined via a commercial available enzyme-linked immunosorbent assay (ELISA) kit (Cell Signaling Tech). BrdU ELISA OD at 405 nm was recorded.

### 2.7. TUNEL staining assay of cell apoptosis

Cells with the indicated TW-37 treatment were fixed by 4% cold formaldehyde (Sigma),followed by incubation with dUTP nick end-labeling (TUNEL) fluorescence dye (Roche, Indianapolis, IN). Cells were then subjected to observation under a confocal microscope (Leica TCS SMD FCS). TUNEL ratio was recorded. For each condition, at least 100 nuclei in five random microscope views (1:100) were analyzed.

### 2.8. Measurement of caspase-3/-9 activities

After TW-37 treatment, 30 μg protein lysates per treatment were incubated with the caspase assay buffer (Alexis Corporation, San Diego, CA) along with the caspase-3 substrate Ac-Asp-Glu-Val-Asp-p-nitroanilide (Ac-DEVD-pNA, Alexis Corporation) or the caspase-9 substrate Ac-Leu-Glu-His-Asp-p-nitroanilide (Ac-LEHD-pNA, Alexis Corporation). After incubation for 30 min, the release of p-nitroanilide (pNA) was detected at 405 nm.

### 2.9. Single-stranded DNA (ssDNA) ELISA assay of apoptosis

The production of ssDNA is known as a key marker of cell apoptosis [17]. After TW-37 treatment, cellular ssDNA content was analyzed via the ELISA format using the commercial available ssDNA kit [18]. The detailed protocol was described previously [18]. The ssDNA ELISA OD at 450 nm was recorded to quantify cell apoptosis.

### 2.10. Western blotting assay

Following treatment, cells were incubated with RIPA lysis buffer (Biyuntian, Wuxi, China). Equal amount of lysate proteins (40 μg per condition) were separated by the SDS-PAGE gels, and were transferred to the polyvinylidenedifluoride (PVDF) membrane (Millipore, Shanghai, China). After blocking, the blot was incubated with designated primary and secondary antibodies. The enhanced chemiluminescence (ECL) reagents (Pierce, Shanghai, China) were added to the blots, and signals were developed under the hyper-X-Ray films. Total gray of each band was quantified using the ImageJ software (NIH), it was always normalized to loading control, β-Tubulin.

### 2.11. Beclin-1 shRNA

Two distinct lentiviral Beclin-1 shRNAs (a/b) as well as the scramble control shRNA were gifts from Dr. Zhu [19].The detailed protocol of lentiviral shRNA infection was described early [19]. After infection, puromycin (5.0 μg/mL) were added to select stable cells for 4–6 days. Knockdown of Beclin-1 in the stable cells was verified by Western blotting assay.

### 2.12. Tumor xenograft assay

The female severe combined immuno-deficient (SCID) *nu/nu* mice (4–5 weeks old, 17–18 grams in weight) were purchased from the Animal Center of The Third Military Medical University. Animals were housed in temperature- and humidity-controlled cages, with free access to water and rodent food on a 12-h light/dark cycle. Mice were injected subcutaneously (*s*.*c*.) with HCT-116 cells (5×10^6^ cells per mouse). When the tumors reached the volume of around 100 mm^3^, the mice were divided into four groups. Mice were treated via intravenous injection (*i*.*v*.) with TW-37 (10 mg/kg body weight, daily for 15 days) or saline vehicle control. TW-37 was always freshly prepared before injection. Estimated tumor volume was calculated by: *π*/6 × larger diameter × (smaller diameter) ^2^. All animal studies were performed in accordance with the standards of ethical treatment approved by the Institutional Animal Care and Use Committee (IACUC) and ERB of The Third Military Medical University. Animals were observed on daily bases. Humane endpoints include a loss of over 15% of body mass, a tumor greater than 1.3 cm, severe fever, vomiting or skin problems or inability to ambulate or rise for food and water. If reaching these endpoints, CO_2_ inhalation was used for euthanasia of animals. All surgical procedures were performed under anesthesia by intramuscular injection of 50% ketamine, 38% xylazine, and 12% acepromazine maleate (0.02 mL). All efforts were made to alleviate suffering.

### 2.13. Statistics analysis

Data were expressed as mean ± standard deviation (SD). A p value, calculated by ANOVA (SPSS16.0), of less than 0.05 was considered statistically significant. The concentrations of reagents applied and the treatment durations were chosen based on published literatures and results from our pre-experiments. IC-50 was calculated also by SPSS software.

## 3. Results

### 3.1. TW-37 inhibits CRC cell survival and proliferation

**Fig 1:**
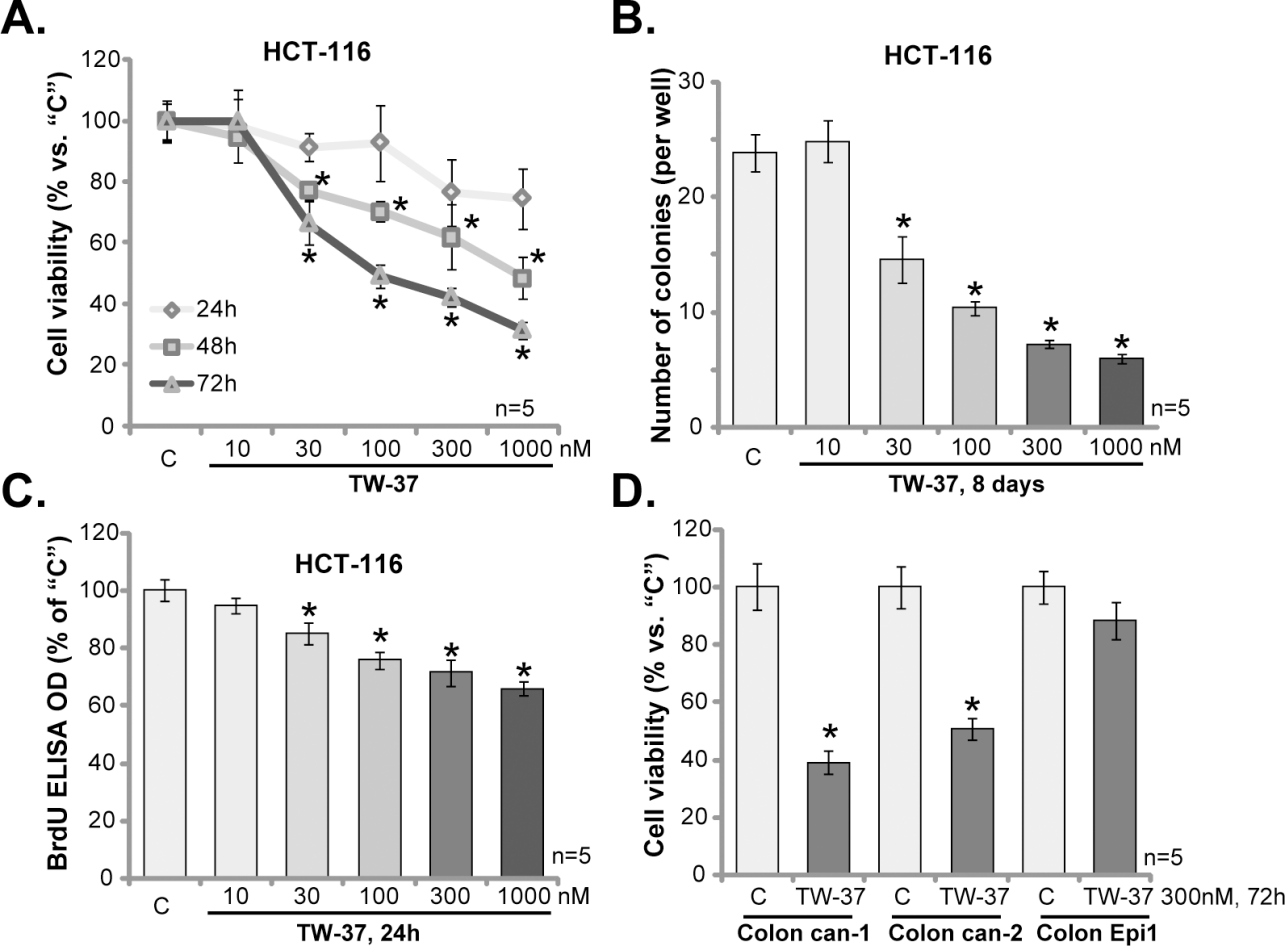
TW-37 inhibits CRC cell survival and proliferation. HCT-116 CRC cells (A-C), the primary human colon cancer cells (two lines, “Colon can-1/2”, D) and the primary human colon epithelial cells (“Colon Epi1”, D) were either left untreated (“C”) or stimulated with TW-37 at applied concentrations (10–1000 nM), cells were further cultured in TW-37-containing-medium for designated time; Cell survival was tested by MTT assay (A and D) or colony formation assay (B, for HCT-116 cells); Cell proliferation was tested by BrdU ELISA assay (C, for HCT-116 cells). Data were presented as mean (n = 5) ± standard deviation (SD). *p<0.05 vs. “C”. Experiments in this figure were repeated five times, and similar results were obtained.

HCT-116 CRC cells were treated with TW-37 at 10–1000 nM. MTT assay was performed to test cell viability. Results in Fig 1A demonstrated that TW-37 efficiently inhibited HCT-116 cell survival. The novel Bcl-2 inhibitor displayed both time- and dose-dependent response in decreasing HCT-116 cell survival (Fig 1A). Treatment with TW-37 for 72 hours was more dramatic than 48 hours in suppressing HCT-116 cells (Fig 1A). The IC-50, or the concentration that led to 50% cell viability reduction, was close to 1000 nM at 48 hours, and 100–300 nM at 72 hours (Fig 1A). The colony formation assay results in Fig 1B showed that TW-37, at 30–1000 nM (treatment renewed every two days), also efficiently decreased the number of viable HCT-116 colonies. The effect of TW-37 was again dose-dependent (Fig 1B).

To study the potential effect of TW-37 on HCT-116 cell proliferation, BrdU incorporation ELISA assay was applied. Quantified results in Fig 1C demonstrated that BrdU ELISA OD was significantly decreased in TW-37 (30–1000 nM)-treated HCT-116 cells, indicating proliferation inhibition. The TW-37’s anti-proliferative activity was again dose-dependent (Fig 1C). Notably, to exclude the possible influence of cytotoxicity, cells were only incubated with TW-37 for 24 hours for analyzing BrdU incorporation (Fig 1C).

Two lines of primary human colon cancer cells were also established. Both were derived from patient cancer tissues and were named as “Colon can-1/2”. MTT assay results in Fig 1D confirmed that treatment with TW-37 (300 nM, 72 hours) was cytotoxic to both lines of primary colon cancer cells. Intriguingly, the very same TW-37 treatment was safe and non-cytotoxic to the primary colon epithelial cells (non-cancerous cells, Fig 1D). These results suggest a selective response of TW-37 in cancerous cells.

### 3.2. TW-37 provokes apoptosis in CRC cells

**Fig 2:**
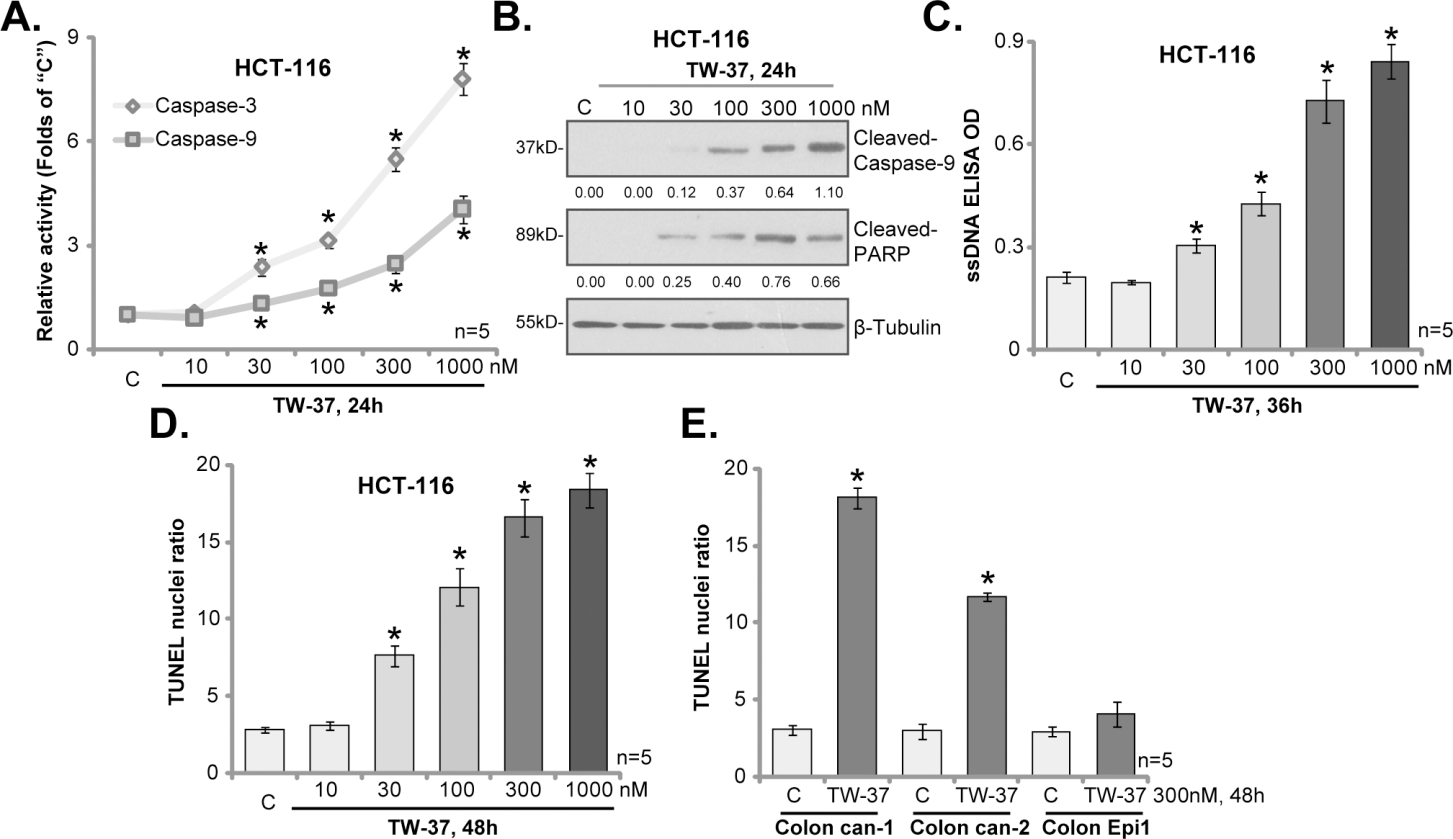
TW-37 provokes apoptosis in CRC cells. HCT-116 CRC cells (A-D), the primary human colon cancer cells (two lines, “Colon can-1/2”, E) and the primary human colon epithelial cells (“Colon Epi1”, E) were either left untreated (“C”) or stimulated with TW-37 at applied concentrations (10–1000 nM), cells were further cultured in TW-37-containing-medium for designated time; Cell apoptosis was tested by the listed assays mentioned in the text. Cleaved-Caspase-9 and Cleaved-PARP were quantified and normalized to β-Tubulin (B). Data were presented as mean (n = 5) ± standard deviation (SD). *p<0.05 vs. “C”. Experiments in this figure were repeated three times, and similar results were obtained.

In order to study the effect of TW-37 on CRC cell apoptosis, the caspase-3/-9 activities were examined. As displayed in Fig 2A, treatment with TW-37 at 30–1000 nM significantly increased the activities of both caspase-3 and caspase-9 in HCT-116 cells. Furthermore, in TW-37-treated HCT-116 cells, cleavages of caspase-9 and poly (ADP-ribose) polymerase (PARP) were observed (Fig 2B), which were followed by the formation of single strand DNA (ssDNA) (Fig 2C). Moreover, TUNEL assay results in Fig 2D showed that the percentage of cells with TUNEL-positive nuclei was dramatically increased following TW-37 (30–1000 nM) treatment. These results confirmed the activation of apoptosis in TW-37-treated HCT-116 cells.

In the two lines of primary colon cancer cells, treatment with TW-37 (300 nM, 48 hours) again induced significant apoptosis activation (TUNEL assay, Fig 2E). TW-37 was however non-apoptotic when added to the colon epithelial cells (TUNEL assay, Fig 2E). Collectively, our results indicate thatTW-37 provokes apoptosis in CRC cells.

### 3.3.Feedback autophagy activation counteracts TW-37

**Fig 3:**
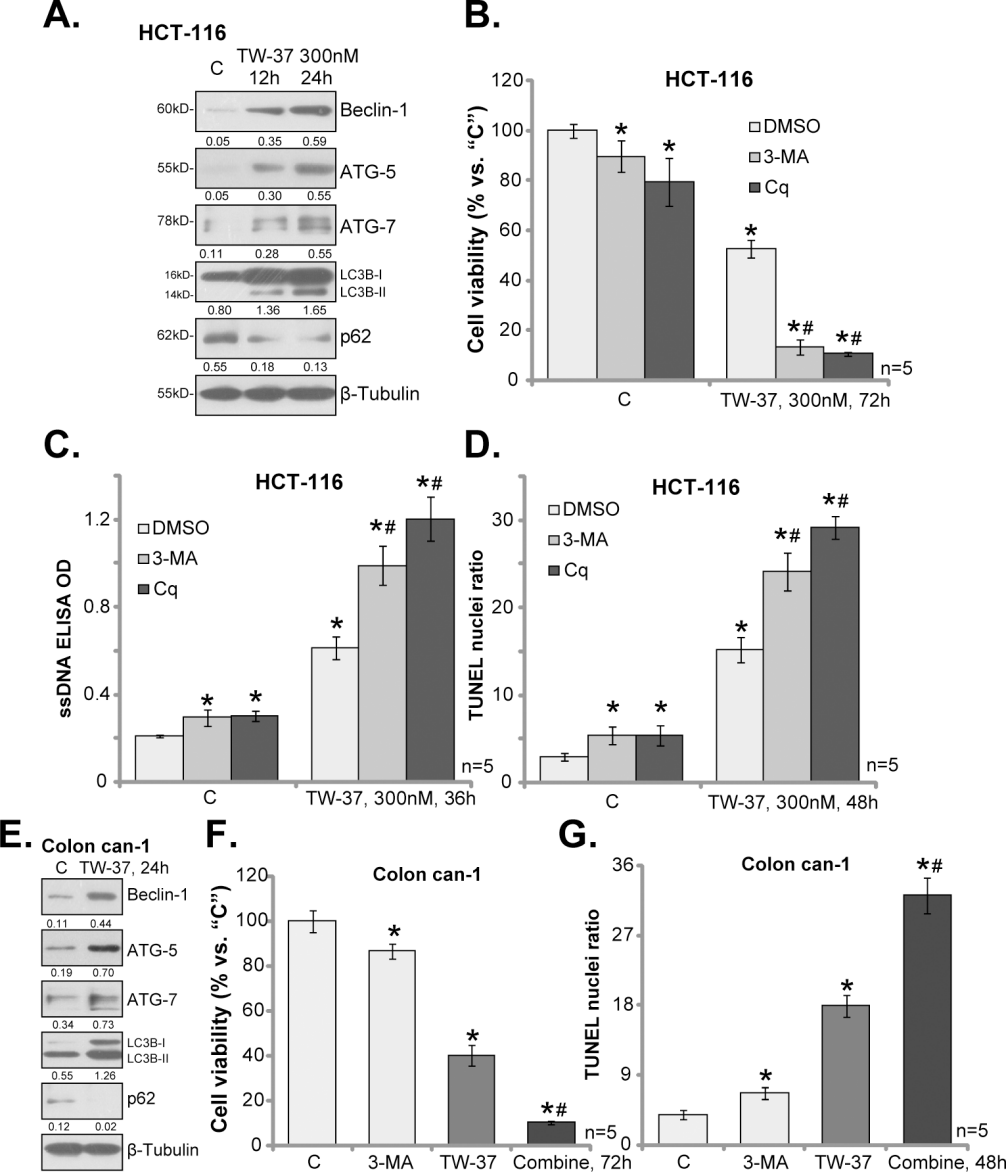
Feedback autophagy activation counteracts TW-37. HCT-116 cells (A-D) and the primary human colon cancer cells (“Colon can-1”, E-G) were treated with TW-37 (300 nM), together with/out3-methyladenine (3-MA, 5 mM) or chloroquine (Cq, 100 μM), cells were further cultured for applied time; Expressions of listed proteins were shown (A and E, data were quantified); Cell survival (B and F) and apoptosis (C, D and G) were tested by the mentioned assays. Data were presented as mean (n = 5) ± standard deviation (SD). *p<0.05 vs. “C” (untreated cells). ^#^ *p*<0.05 vs. TW-37 only group. Experiments in this figure were repeated three times, and similar results were obtained.

One major objective of this study is to identify possible TW-37’s resistance factors. Exiting evidences have suggested that autophagy could be activated following treatment of various anti-cancer agents, which counteracts cancer cell death/apoptosis[20,21,22,23]. Meanwhile, several established Bcl-2 inhibitors, *i*.*e*. ABT-737, were shown to provoke cytoprotective autophagy[24,25]. Western blotting assay results in Fig 3A showed that TW-37 (300 nM) treatment in HCT-116 cells time-dependently induced expressions of autophagy-related proteins, including Bcelin-1, ATG-5, ATG-7 and LC3B-II, but degradation of p62. These results implied an autophagy flux response in TW-37-treated cells, suggesting autophagy activation[21].

To study the potential function of autophagy in TW-37-induced activity against HCT-116 cells, pharmacological strategy was first applied. Two well-established autophagy inhibitors, including 3-methyladenine (3-MA) [26] or chloroquine[27], were applied. As displayed in Fig 3B, co-treatment with each autophagy inhibitor significantly exacerbated TW-37-induced HCT-116 cell viability reduction. Furthermore, TW-37-induced apoptosis activation was also sensitized in the presence of 3-MA or Cq (Fig 3C and 3D). Notably, treatment with 3-MA or Cq alone only induced minor viability reduction and apoptosis in HCT-116 cells.

In the primary colon cancer cells (“Colon can-1”), treatment of TW-37 (300 nM, 24 hours) also induced above change in the expressions of the autophagy proteins (Fig 3E). More importantly, TW-37-induced viability reduction (Fig 3F) and apoptosis (Fig 3G) were again potentiated with co-treatment of 3-MA, the autophagy inhibitor. Collectively, these results suggest that TW-37 activates feedback autophagy in CRC cells, and pharmacological inhibition of autophagy significantly sensitizes TW-37-induced killing of CRC cells.

### 3.4. Beclin-1 shRNA sensitizes TW-37-induced killing of HCT-116 cells

**Fig 4:**
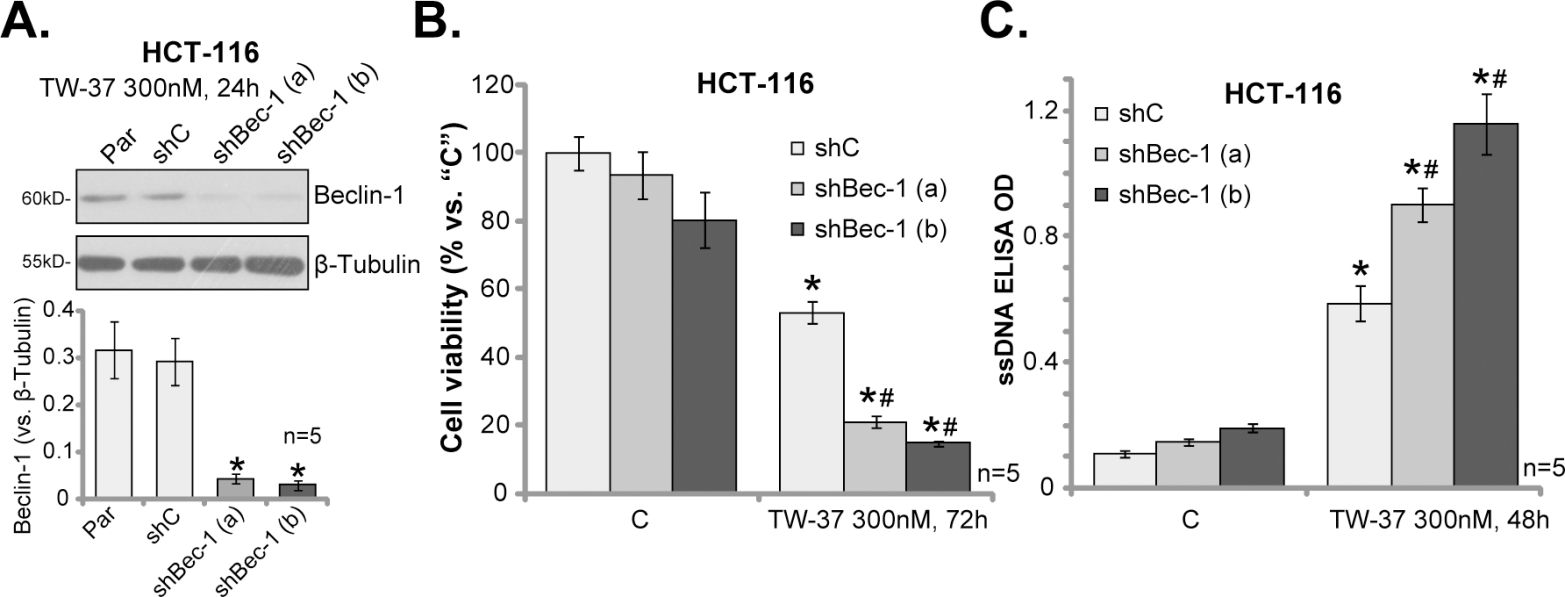
Beclin-1 shRNA sensitizes TW-37-induced killing of HCT-116 cells. Stable HCT-116 cells, expressing lentiviral Beclin-1 shRNA [two distinct ones with non-overlapping sequences, “shBec-1 (a)/(b)”], scramble non-sense control shRNA (“shC”), as well as the parental control cells (“Par”), were treated with/out TW-37 (300 nM) for applied time; Expression of listed proteins was shown (A, data were quantified), Cell survival (MTT assay, B) and apoptosis (ssDNA ELISA assay, C) were also tested. Data were presented as mean (n = 5) ± standard deviation (SD). *p<0.05 vs. “C”. ^#^ *p*<0.05 vs. “shC” cells. Experiments in this figure were repeated four times, and similar results were obtained.

Beclin-1 is a key autophagy-associated protein, which is required for the formation of autophagy vehicles[28]. The above results have demonstrated that TW-37 induced Beclin-1 protein expression in HCT-116 cells. We next utilized genetic strategy to interfere Beclin-1 expression. As described, two distant lentiviral Beclin-1 shRNAs were introduced to HCT-116 cells. The two shRNAs each efficiently downregulated Beclin-1 in TW-37-treated HCT-116 cells (Fig 4A). Consequently, TW-37-induced viability reduction (Fig 4B) and apoptosis activation (Fig 4C) were significantly potentiated with Beclin-1 shRNA. These shRNA results further implied a negative role of autophagy in TW-37-meidated anti-CRC cell activity.

### 3.5. TW-37 intravenous injection inhibits HCT-116 tumor growth *in vivo*

**Fig 5:**
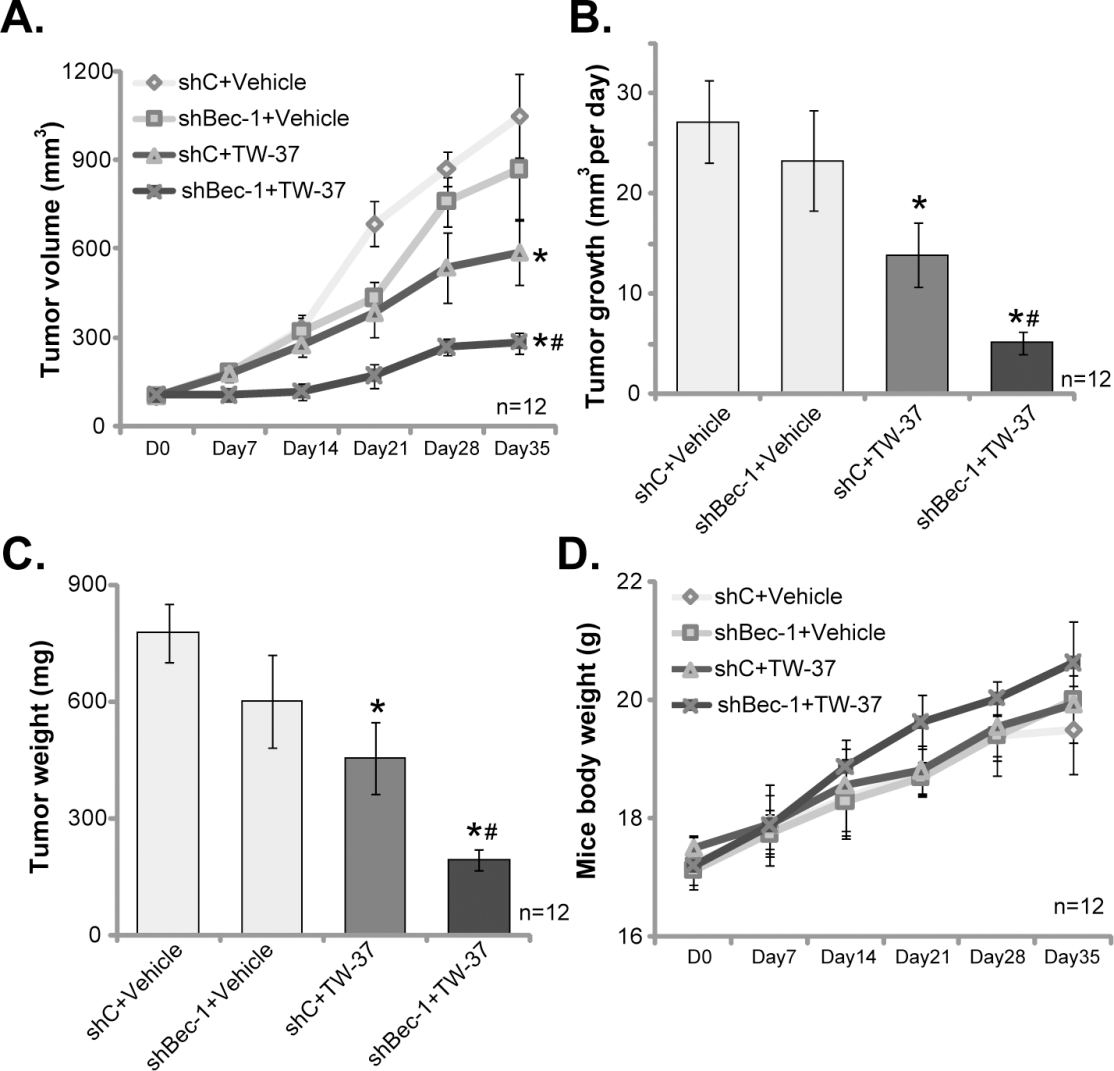
TW-37 intravenous injection inhibits HCT-116 tumor growth *in vivo*. Stable HCT-116 cells, expressing lentiviral Beclin-1 shRNA [“shBec-1 (a)”] or scramble non-sense control shRNA (“shC”), were *s*.*c*. injected to the flanks of SCID mice. When the tumor volumes were about 100 mm^3^, mice were treated via intravenous injection of TW-37 (10 mg/kg body weight, daily for 15 days) or saline (“Vehicle”); Tumor volumes (A) and mice body weights (D) were recorded every 7 days for 35 days; Estimated daily tumor growth (in mm^3^ per day) was also calculated (B); At Day-35, mice were euthanized, tumors were removed and weighted individually (C). * *p*< 0.05 vs. “shC+Vehicle” group. ^#^*p*< 0.05 vs. “shC+TW-37” group.

At last, HCT-116 cells, expressing scramble non-sense control shRNA (“shC”), were *s*.*c*. injected to the flanks of the SCID mice to establish xenograft tumor model. The *in vivo* activity of TW-27 was tested via intravenous (*i*.*v*.) injection of TW-37 to the HCT-116-tumor bearing mice. Weekly tumor growth curve results in Fig 5A demonstrated that TW-37 injection (10 mg/kg body weight, daily for 15 days) significantly inhibited HCT-116 xenograft growth in SCID mice. Estimated daily tumor growth reduced from 27.13± 4.09mm^3^ per day (in vehicle control mice) to 13.89 ± 3.23 mm^3^ per day after TW-37 injection (Fig 5B). The weight of the tumors (At Day-35) in TW-37-treated mice was also lighter than that of the control mice (Fig 5C). Thus, TW-37 *i*.*v*. injection inhibited HCT-116 tumor growth *in vivo*.

To study the role of autophagy in TW-37’s activity *in vivo*, the stable HCT-116 cells with Beclin-1 shRNA (“sh-Bec-1”) were also inoculated to the SCID mice, establishing Beclin-1-silenced tumors. As demonstrated, the growth of Beclin-1-silenced tumors was only slightly slower than the control tumors (Fig 5A–5C). Remarkably, TW-37-induced activity *in vivo* was significantly potentiated against Beclin-1-silenced tumors (Fig 5A–5C). Estimated tumor volume (Fig 5A), daily tumor growth (Fig 5B) and the tumor weight (Fig 5C) were most significantly inhibited in TW-37-treated Beclin-1-silenced tumor group (“shBec-1+TW-37”). These results suggested that autophagy inhibition, via silencing Beclin-1, might also potentiate TW-37-induced anti-HCT-116 tumor activity (Fig 5A–5C). Notably, there was no significant difference in mice body weights between the groups (Fig 5D). We also failed to detect any apparent toxicities among the tested animals. Thus, these mice were well-tolerated to the TW-37 regimens.

## 4. Discussion and conclusion

The anti-apoptosis Bcl-2 family proteins, including Bcl-2, Mcl-1, Bcl-xL, are often over-expressed in CRC [29,30] and many other malignancies [8,9]. They are important for CRC progression and chemo-/ratio-resistance[8,9]. TW-37 is a newly-developed small-molecule Bcl-2 inhibitor, it targets multiple members of anti-apoptosis Bcl-2 proteins[8,9]. In the current study, treatment with TW-37 at only nM concentration inhibited survival and proliferation of both HCT-116 cells and primary human colon cancer cells. Meanwhile, TW-37 induced activation of caspase-3/9 and apoptosis in the CRC cells. *In vivo*, intravenous injection of TW-37 inhibited HCT-116 tumor growth in mice. Our results suggest that targeting Bcl-2 family protein by TW-37 could be a fine strategy to inhibit CRC cells.

Autophagy begins with the formation of the double membrane vesicle structures in the cytoplasm, named autophagosomes[31,32], those are responsible for degrading organelles and other cytoplasmic contents via acidic lysosomal hydrolases [31,32]. The process will provide nutrition and energies for cell to survive[31,32]. Dysregulation of autophagy has been detected in different types of cancers, which is associated with cancer cell progression and chemo-resistance [32,33]. Meanwhile, multiple cancer-killing agents of different mechanisms of action were demonstrated to induce feedback and cytoprotective autophagy activation[32,33]. Reversely, autophagy inhibition via genetic or pharmacologic methods, could significantly sensitize the anti-cancer activity by the agents [20,21].

One protein that is vital for autophagosome formation and autophagy progression is Beclin-1, which is the mammalian ortholog of yeast ATG6 [28]. Beclin-1 associates with a number of co-factors to form autophagosomes[28]. Interestingly, Beclin-1 could form a complex with Bcl-2 family proteins. On the other hand, Bcl-2 inhibition might then be able to cause Beclin-1 upregulation and cell autophagy [24,25,34]. Pedro *et al*., showed that ABT-737, a Bcl-2/Bcl-xL inhibitor [35], induced Bcl-2-Beclin-1 complex dissociation, causing Beclin-1 upregulation and subsequent autophagy induction[25]. Yao *et al*., demonstrated that Bcl-2 inhibition by ABT-737 led to activation of cyto-protective autophagy, which was dependent on Beclin-1 upregulation[24]. On the other hand, autophagy inhibitors or Beclin-1 knockdown significantly increased the sensitivity of ABT-737 in hepatocellular carcinoma (HCC) cells [24].

In the current study, TW-37 treatment also induced feedback autophagy activation in CRC cells. Autophagy was evidenced by expressions of autophagy-related proteins (Bcelin-1, ATG-5, ATG-7 and LC3B-II), but degradation of p62. Remarkably, autophagy inhibitors (3-MA and Cq) or Beclin-1 knockdown by targeted-shRNA dramatically sensitized TW-37-induced killing of CRC cells. TW-37-meiated anti-tumor activity *in vivo* was also potentiated against Beclin-1-silenced tumors. Therefore, feedback autophagy activation appears to be a primary chemo-resistance factor of TW-37in CRC cells. Inhibition of autophagy activation, on the other hand, sensitizes TW-37’s anti-CRC cell activity.
